# *Euphorbia tirucalli* modulates gene expression in larynx squamous cell carcinoma

**DOI:** 10.1186/s12906-016-1115-z

**Published:** 2016-05-21

**Authors:** Gabriela Bueno Franco-Salla, Janesly Prates, Laila Toniol Cardin, Anemari Ramos Dinarte dos Santos, Wilson Araújo da Silva Jr, Bianca Rodrigues da Cunha, Eloiza Helena Tajara, Sonia Maria Oliani, Flávia Cristina Rodrigues-Lisoni

**Affiliations:** Department of Biology, Institute of Biosciences, Letters and Science - IBILCE/UNESP, São José do Rio Preto, SP Brazil; Department of Clinical Medical, Foundation Blood Center of Ribeirão Preto, Faculty of Medicine of Ribeirão Preto, University of São Paulo - FCFRP/USP, Ribeirão Preto, SP Brazil; Department of Molecular Biology, Faculty of Medicine of São José do Rio Preto - FAMERP, São José do Rio Preto, SP Brazil; Department of Biology and Animal Science, Faculty of Engineering of Ilha Solteira - FEIS/UNESP, Av. Brasil, 56, CEP: 15385-000 Ilha Solteira, São Paulo Brazil

**Keywords:** Differential gene expression, Cell culture, Subtractive hybridization, Quantitative PCR

## Abstract

**Background:**

Some plants had been used in the treatment of cancer and one of these has attracted scientific interest, the *Euphorbia tirucalli* (*E. tirucalli*), used in the treatment of asthma, ulcers, warts has active components with activities scientifically proven as antimutagenic, anti-inflammatory and anticancer.

**Methods:**

We evaluate the influence of the antitumoral fraction of the *E. tirucalli* latex in the larynx squamous cell carcinoma (Hep-2), on the morphology, cell proliferation and gene expression. The Hep-2 cells were cultivated in complete medium (MEM 10 %) and treated with *E. tirucalli* latex for 1, 3, 5 and 7 days. After statistically analyzing the proliferation of the tested cells, the cells were cultivated again for RNA extraction and the Rapid Subtractive Hybridization (*RaSH*) technique was used to identify genes with altered expression. The genes found using the *RaSH* technique were analyzed by Gene Ontology (GO) using Ingenuity Systems.

**Results:**

The five genes found to have differential expression were validated by real-time quantitative PCR. Though treatment with *E. tirucalli* latex did not change the cell morphology in comparison to control samples, but the cell growth was significantly decreased. The *RaSH* showed change in the expression of some genes, including *ANXA1, TCEA1, NGFRAP1, ITPR1* and *CD55*, which are associated with inflammatory response, transcriptional regulation, apoptosis, calcium ion transport regulation and complement system, respectively. The *E. tirucalli* latex treatment down-regulated *ITPR1* and up-regulated *ANXA1* and *CD55* genes, and was validated by real-time quantitative PCR.

**Conclusions:**

The data indicate the involvement of *E. tirucalli* latex in the altered expression of genes involved in tumorigenic processes, which could potentially be applied as a therapeutic indicator of larynx cancer.

**Electronic supplementary material:**

The online version of this article (doi:10.1186/s12906-016-1115-z) contains supplementary material, which is available to authorized users.

## Background

The head and neck squamous cell carcinoma (HNSCC) represents malignant tumors arising primarily in the oral cavity, tongue, floor of the mouth, tonsils, pharynx and larynx. Worldwide HNSCC is the fifth most common cancer and it accounts for about 6 % among all cancers [1]. This tumor originates from various anatomic structures including the craniofacial bones, soft tissues, salivary glands, skin, and mucosal membranes [2].

The main risk factors for larynx squamous cell carcinoma (LSCC) are smoking and alcohol consumption [3]. Family history is another important risk factor for this cancer, suggesting that genetic factors may contribute to susceptibility of this disease [4]. Others aspects that can modify cancer risk factors of the head and neck are the polymorphisms in drug metabolizing enzymes (DMEs) and prevalence of variant genotypes of cytochrome P450 (CYP) 1A1, 1B1, 2E1 or glutathione-S-transferase M1 (null). Furthermore, strong associations of the polymorphic genotypes of DMEs with cases of pharyngeal and oral cavity cancer who were tobacco chewers or alcohol users demonstrate that gene-environment interactions may explain differences in genetic susceptibility for cancers of the oral cavity, pharynx and larynx [5].

Some authors have reported that DNA alterations are responsible for the onset of carcinogenesis that usually occur in genes involved with proteins regulating cell cycle, cell growth, and differentiation. These are classified as oncogenes and tumor suppressor genes [6]. In HNSCC, the oncogenes *H-ras*, *c-myc*, *EGFR* and *TGF-β* are often activated and the tumor suppressor genes such as *TP53*, *p16*, *FHIT*, *PTEN*, *Rb* and *APC* are inactivated [7, 8].

Other factors that may contribute to LSCC include diet, oral hygiene, body mass, environmental pollution, certain working conditions associated with industries such as metallurgy and petrochemical and infections by human papillomavirus - HPV, particularly types 16 and 18 and Epstein-Barr virus which appear to be associated with the development of more aggressive tumors [7, 9–11]. The treatment for this neoplasm is defined depending on the type of cell, degree of differentiation, location, extent, presence of lymph node metastases, and macroscopic characteristics with bone and muscle tumor involvement. Conventional methods used for treatment are surgery, chemotherapy, and radiation therapies, but all these methods are considered highly invasive. Surgery can cause irreversible aesthetic injuries with possible functional alterations [12]. However, some plants have been used in the treatment of cancer as alternative therapies in an attempt to soften the damage caused by conventional methods. The *Euphorbia tirucalli* (*E. tirucalli*), popularly known as aveloz, which is used in the herbal treatment of asthma, ulcers, warts and tumors in general has attracted scientific interest [13, 14].

Originating from Africa, *E. tirucalli* belonging to the family Euphorbiaceae, was subsequently introduced in other countries, it has some compounds that have been scientifically proven to have biological activities such as antiviral, anticancer for breast, antibacterial, anti-inflammatory, antimutagenic, antitumor and larvicidal [13, 15–18]. In addition, some authors have reported that a lectin isolated from the latex of *E. tirucalli*, called Eutirucallin showed biological activities such as neutrophils recruitment and cytokine production by macrophages [19]. An alternative that justifies its antitumorigenic potential is antimutagenicity. Substances with antimutagenic potentials increase the efficiency of repair mechanisms thereby acting as protective agents that decrease the frequency of DNA damage while controlling the disorderly proliferation of tumor cells [17].

The euphol (tetracyclic triterpene alcohol isolated from the latex of *E. tirucalli*) showed anticancer effects, inhibiting the growth of human gastric cancer cells and tumor development, demonstrating its great value and potential for use in disease therapy [20]. Anti-inflammatory properties of euphol was observed in skin diseases, because it markedly inhibited TPA (12-O-tetradecanoilphorbol-13-acetato)-induced inflammatory response, demonstrating that euphol can be used as an alternative therapy in skin diseases [21].

To investigate the effects of *E. tirucalli* in LSCC, we evaluated the influence of this herbal medicine on the morphology, cell proliferation and gene expression in Hep-2 cells. We also identified the signaling pathways where genes modulated by *E. tirucalli* participate, which are important for tumorigenic and the inflammatory process.

## Methods

### Hep-2 culture conditions

The Hep-2 cell line originally established from an epidermoid carcinoma of the larynx (ATCC, Rockville, Maryland, USA), was obtained and seeded at a density of 1 × 10^6^ cells/ml per 75 cm^2^ culture flask (Corning, NY, USA). Minimum Essential Medium (MEM - Cultilab, Campinas, SP, Brazil) was used at pH 7.5 and supplemented with 10 % fetal calf serum (Cultilab), 1 % non-essential amino acids and 1 % antibiotic/antimycotic (Invitrogen Corporation, Carlsbad, CA, USA) and cultured at 37 °C under an atmosphere of 5 % CO_2_.

### Pharmacological treatments

The Hep-2 cells were subdivided into three groups: a) control that was not manipulated, b) the group that was submitted to the addition of vehicle (ethanol) and c) an experimental group where *E. tirucalli* latex was added at concentrations of 0.25 to 250 μg/ml. The experiments were conducted for periods of 1, 3, 5, and 7 days to determine the statistically significant time for further use in the methodology, for all the three groups.

The sap from E. tirucalli was initially extracted with hexane through an incision in the trunk and branches of the adult plant, and the resulting precipitate was extracted with n-butanol. The active fraction of this latex, diluted in this polar solvent, was used to treat the cells. In this butanol fraction of the *E. tirucalli* latex, ingenol-3-hexanoate was used to obtain a useful composition for the treatment of disease. Synthesis and modifications were done by Kyolab Laboratories (Campinas, Brazil), by Dr. Luiz Francisco Pianowski.

Separation of the butanol fraction by HPLC (High Performance Liquid Chromatography) allowed the compounds present to be taken off in group scales, mainly by size. In a column chromatography separation with silica gel Sephadex G75, using a mixture of hexane:ethyl acetate (0 to 100 %), eight fractions were separated from the butanol fraction of the latex extract. But one of them show the best presence of components with higher polarity, with solubility and affinity characteristics with the substrate, then this fraction was submitted to tests to verify its anticancer action, as disclosed in the “patent of the active fraction of a polar solvent extract from the latex of Euphorbiaceae plants” (Additional file 1) and results in decreased of growth of seven cancer cell lines [22].

### Growth curve

Hep-2 cells were seeded in triplicate at a density of 5 × 10^4^ cells in plastic 6-well plates and divided in three groups. The control group was grown on complete medium, the other groups were submitted to the addition of vehicle (ethanol), whereas *E. tirucalli* latex was added to the experimental group. Twenty four (24) hours later, when the cells had already adhered, Hep-2 cultures were incubated with serum free medium (MEM 0 %). After an additional 24 h, the control group was maintained in complete medium without any pharmacological manipulation, whereas vehicle (ethanol) was added to the other groups and *E. tirucalli* latex was added to the experimental group.

The cell morphology was observed on each day. The cytotoxic effects of the *E. tirucalli* on the proliferation of Hep-2 cells were investigated in three experimental groups at 1, 3, 5 and 7 days. The cells were harvested, stained with trypan blue and counted using the Countess® Automated Cell Counter (Invitrogen®). Significant differences between the groups were determined by one-way analysis of variance (*GraphPad Prism 5*) and statistical significance was assumed when *P* <0.05. The same experiment was repeated twice.

### RNA extraction for Rapid Subtraction Hybridization (RaSH) and real time PCR experiments

Hep-2 cells were seeded at a density of 1 × 10^6^ cells/ml in 75 cm^2^ culture flasks in complete medium (control group), vehicle (ethanol) was added and they were incubated with *E. tirucalli* latex for 3 days (experimental group). The RNA from Hep-2 cells (three groups) was extracted using Trizol [23] following treatment with DNase (Invitrogen). cDNA synthesis was performed using a High Capacity cDNA Archive kit (Applied Biosystems, Foster City, CA, USA) according to the manufacturer’s instructions.

### Rapid Subtraction Hybridization (RaSH)

The *RaSH* technique [24] was performed with two subtractions. The first was termed “Sub I”, in which the Hep-2 control cells were referred to as *tester* and the cells treated with *E. tirucalli* latex were termed *driver*. In the second subtraction, “Sub II,” the opposite test was performed.

The first cDNA strand was obtained by reverse transcriptase with 2 μl of oligo (dT) (50 mM) and 2 μl of 10 mM dNTPs at 65 °C for 5 min. The samples were immediately placed on ice for 1 min and 2.0 μl of 0.1 mM DTT, 1 μl of 40 U/μl RNA-OUT and 4.0 μl of 5X First-Strand Buffer [250 mM Tris-HCl (pH 8,3), 375 mM KCl, 15 mM MgCl_2_] were added. The samples were incubated at 42 °C for 2 min and 1.0 μl of Superscript™ II reverse transcriptase (RT) (200 U/μl) was added. The samples were incubated again at 42 °C for 50 min and then at 70 °C for 15 min. Segments of β-actin cDNA were also amplified to verify the quality of these reactions.

The second strand of cDNA was synthesized in reactions containing 30 μl of 5X Second Strand Buffer, 3 μl of 10 mM dNTPs, 1 μl of DNA ligase (10 U/μl), 4 μl of DNA Pol I (10 U/μl) and 1.0 μl of RNase H (2 U/μl). The samples were incubated for 2 h at 16 °C before the addition of 2 μl of T4 DNA Pol (5 U/μl) and further incubation for an additional 5 min. The action of the enzyme T4 DNA Pol was inhibited by the addition of 10 μl of 0.5 M EDTA. Phenol/chloroform extraction was used to purify the sample and the pellet was resuspended in 45 μl of water. The cDNA was used in amplification reactions for segments of GAPDH and was subsequently digested with 2 μl of MboI enzyme (10 U/μL) and incubated for 1 h at 37 °C. Finally, over 1 μl of this enzyme was added to the reaction, and the samples were incubated overnight at 37 °C.

A volume of 4.1 and 4.5 μl, of the adapters XDPN-14 5′-CTGATCACTCGAGA and XDPN-12 5′-GATCTCTCGAGT (Sigma Chemical, final concentration 10 mM) was added to the cDNA respectively, 8 μl of 5X T4 DNA ligase buffer Invitrogen©, heated at 55 °C for 1 min, and cooled down to 14 °C within 1 h was also added. The cDNA received 3 μl (9 U) of T4 DNA ligase (3 U/μl), ligation was carried out overnight at 14 °C. After phenol/chloroform extraction and ethanol/glycogen precipitation, the mixtures were diluted to 100 μl with TE buffer (10 mM Tris/1 mM EDTA); 40 μl of the mixtures were used for PCR amplification.

The PCR mixtures were set up using 10 μM XPDN-18 5′-CTGATCACTCGAGAGATC, 0.4 mM dNTPs, 10 × PCR buffer, 1.5 mM MgCl_2_ and 1U Taq DNA polymerase (Invitrogen). Thermocycler conditions were one cycle at 72 °C for 5 min, followed by 25 cycles of 94 °C for 1 min, 55 °C for 1 min, 72 °C for 1 min, ending in a final extension at 72 °C for 3 min. Ten (10 μg) micrograms of purified PCR product (tester) was digested with 10 U/μl XhoI (Invitrogen) for 6 h at 37 °C and followed phenol/chloroform extraction and ethanol precipitation.

One-hundred nanograms of the tester cDNA were mixed with 5 μg of the driver cDNA in hybridization solution (0.5 M NaCl, 50 mM Tris/HCl, SDS 2 % and 40 % formamide) and, after heating at 99.9 °C for 5 min, they were incubated at 42 °C for 1 h, and 42 °C for 48 h. After extraction and precipitation, the hybridization mixture (1 μg) was ligated with XhoI-digested pZero plasmid and transformed into competent bacteria. Bacterial colonies were picked and used as DNA template for PCR. Clones were sequenced using an automated DNA sequence and homologies sequences were searched using the BLAST program. Gene ontology (GO) annotation was used for the functional classification of up- and down-regulated genes using terms from Gene Ontology database.

The genes were further analyzed through Ingenuity Pathway (IPA - Ingenuity Systems©) to relate the differentially expressed genes with biological functions and processes in which these genes are involved and evaluate the main networks of interaction between them.

### Quantitative PCR - RT-qPCR

The RNA from control, vehicle (ethanol) and *E. tirucalli* latex treated Hep-2 cells was extracted and cDNA synthesis was performed as described above.

Five differentially expressed genes were selected for validation by quantitative real time PCR experiments according to their direct or indirect involvement in tumorigenesis. Their expression was checked in treated samples relative to matched non-treated samples.

The primers were manually designed with: 19–23 bp length, 30–80 % GC content and a short amplicon size (80–120 bp). Their sequences are shown in Table 1. Real-time PCR was performed in triplicate using a 7500 Fast Real-Time PCR System (Applied Biosystems). Reaction mixture consisted of a 20 μl volume solution containing 10 μl of Power SYBR Green PCR Master Mix (Applied Biosystems).

**Table 1:** Primers for the selected genes. *ANXA1, ITPR1, CD55, NGFRAP1* and *TCEA1* primers to quantitative PCR validation

Primer	Sequence
ANXA1 antisense	5′ TAAGGGTGACCGATCTGAGG 3′
ANXA1 sense	5′ ACGTCTGTCCCCTTTCTCCT 3′
ITPR1 antisense	5′ GCTCTATGAGCAGGGGTGAG 3′
ITPR1 sense	5′ GGAACACTCGGTCACTGGAT 3′
*CD55* antisense	5′ CAGCACCACCACAAATTGAC 3′
*CD55* sense	5′ TGCTCTCCAATCATGGTGAA 3′
NGFRAP1 antisense	5′ GGGGGAGCTCTCTAATCACC 3′
NGFRAP1 sense	5′ AAAGAAAACAGCGGGAATCA 3′
TCEA1 antisense	5′ AGCTGAAAGAGATGCGGAAA 3′
TCEA1 sense	5′ TGCCACATGTGAACAAGTCA 3′

Reaction mixture consisted of a 20 μl volume solution containing 100 and 500 ηg of Power SYBR Green PCR Master Mix (Applied Biosystems), 100 nM of each primer and 100 ng cDNA (control and treated with *E. tirucalli* latex). The PCR conditions were 95 °C for 10 min followed by 40 cycles of 95° for 15 s and 60° for 1 min. Melting curve analysis was performed for each gene to check the specificity and identity of the RT-PCR products. For each primer set, the efficiency of the PCR reaction (linear equation: y = slope + intercept) was measured in triplicate on serial dilutions of the same cDNA sample. The PCR efficiency (E) was calculated by the formula *E* = [10^(-1/slope)^] and ranged from 1.96 to 2.02 in the different assays.

Two control genes (*GAPDH, ACTB*) were used as internal standards. The relative expression ratio (fold change) of the target genes was calculated according to Pfaffl [25]. Statistical analysis was performed by a two-tailed unpaired *t* test using GraphPad prism software.

## Results

### Cellular morphology

**Fig. 1:**
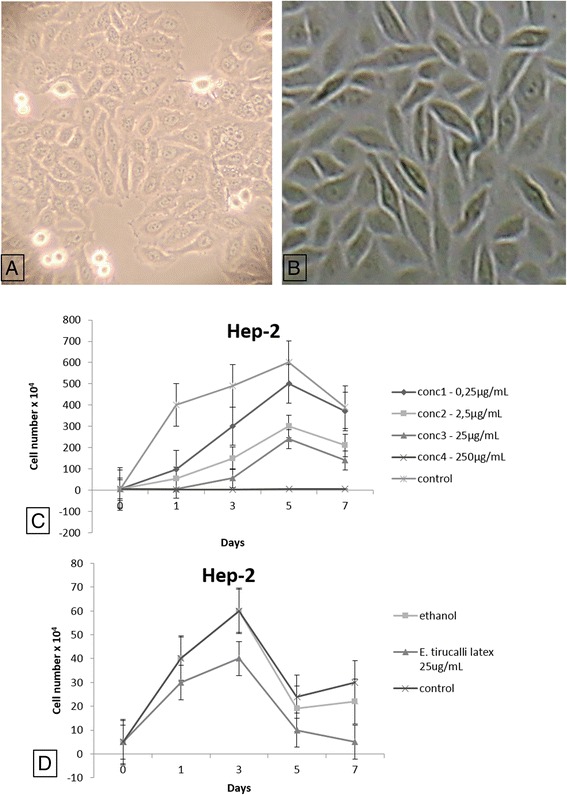
Morphological analysis and growth curve of *E. tirucalli* latex treatment in Hep-2 cell line. Hep-2 cells were seeded in complete MEM 10 % at a concentration of 5×10^4^ per well (6 well plate). The cell morphology observed in (**a**) the control group, characterized by a monolayer of nucleated cells was not altered in (**b**) the *E. tirucalli* latex [25 μg/mL] treated group for 3 days. 400× magnification. The growth curve observed to Hep-2 cells treated with (**c**) *E. tirucalli* latex [0.25, 2.5, 25, 250 μg/ml] for 1, 3, 5 and 7 days and (**d**) ethanol (vehicle) and *E. tirucalli* latex [25 μg/ml] for 1, 3, 5 and 7 days. Assays were performed in triplicate. Graph x = time (hours) and y = number of cells × 10^4^. *P* values <0.05 were significant

Hep-2 cells were initially cultured and observed by phase contrast microscopy. These cells have a fusiform appearance and central core, with multiple nucleoli and a eucromatic position, which are characteristics of a cell with high metabolic activity. In cells treated with *E. tirucalli* latex [25 μg/ml], there was no morphological change, and cytoplasmic processes were more clearly observed due to reduced cell proliferation (Fig. 1a and b).

### Proliferation assay

In the proliferation assay (Fig. 1c), it was possible to observe the growth inhibitory effect under treatment with *E. tirucalli* latex, using the GraphPad Prism 5 (GraphPad Software). *E. tirucalli* was shown to decrease the proliferation in treated cells in comparison to the control group. Statistical analysis showed that these differences were most significant (*P* < 0.05) at a concentration of 25 μg/ml *E. tirucalli* and a culture time of 3 days.

We also observed, using the same statistical test, that the addition of ethanol reduced cell proliferation, however, they did not exhibit any statistically significant difference compared to control group (Fig. 1d), confirming that the reduced growth of Hep2 cells was due to *E. tirucalli* and not its diluent’s (ethanol), (Additional files 2 and 3).

### Genes identified using the RaSH approach

Rapid subtractive hybridization (*RaSH*) compares two distinct experiments aiming to find differential gene expression. *RaSH* was performed on Hep-2 cells from the control group and those treated with *E. tirucalli* latex for 3 days and 85 clones were isolated and sequenced. By screening these sequences through GenBank (*Blast*) and by rigorous classification and selection, 19 genes were found to be differentially expressed, and two libraries of genes (Sub I and Sub II) were acquired. Fifteen genes of the “Sub I” library exhibited changes in expression levels in the control cells, whereas four genes of the “Sub II” library changed in expression under treatment with *E. tirucalli* latex. Therefore, it was expected that the gene expression of one library would be lower than that of the other one. The libraries of the genes obtained along with their different functions and processes are described in the Gene Ontology (GO) databases.

IPA was used to further analyze the 19 differentially expressed genes, which were especially associated with tumorigenesis, apoptosis, cell proliferation, cell cycle, DNA repair, complement activation and inflammatory response (Table 2). Moreover, some of these genes were chosen because they form a network of important interactions with other genes such as *NF-kB*.

**Table 2:** Genes selected by *RasH* in the subtractive library I expressed in Hep-2 cell line. NM is the access number in GenBank (www.ncbi.nlm.nih.gov)

NM	Name	Abbreviation	Chromosomal location	Functions and processes
NM_001099952.2	Homo sapiens inositol 1,4,5-trisphosphate receptor, type 1	ITPR1	3p26.1	Protein binding; calcium ion transmembrane transporter activity; intrinsic apoptotic signaling pathway and signal transduction.
NM_000700.1	Homo sapiens annexin A1	ANXA1	9q21.13	Calcium ion binding; regulation of cell proliferation; inflammatory response; cell surface receptor signaling pathway; negative regulation of apoptotic process.
NM_006756.2	Homo sapiens transcription elongation factor A (SII), 1	TCEA1	8q11.2	DNA repair; positive regulation of transcription from RNA polymerase II promoter and gene expression.
NM_000574.3	Homo sapiens *CD55* molecule, decay accelerating factor for complement	*CD55*	1q32	Enzyme inhibitor activity; elevation of cytosolic calcium ion concentration and regulation of complement activation.
NM_206917.1	Homo sapiens nerve growth factor receptor (TNFRSF1) associated protein 1	NGFRAP1	Xq22.2	Protein binding; induction of apoptosis.

**Fig. 2:**
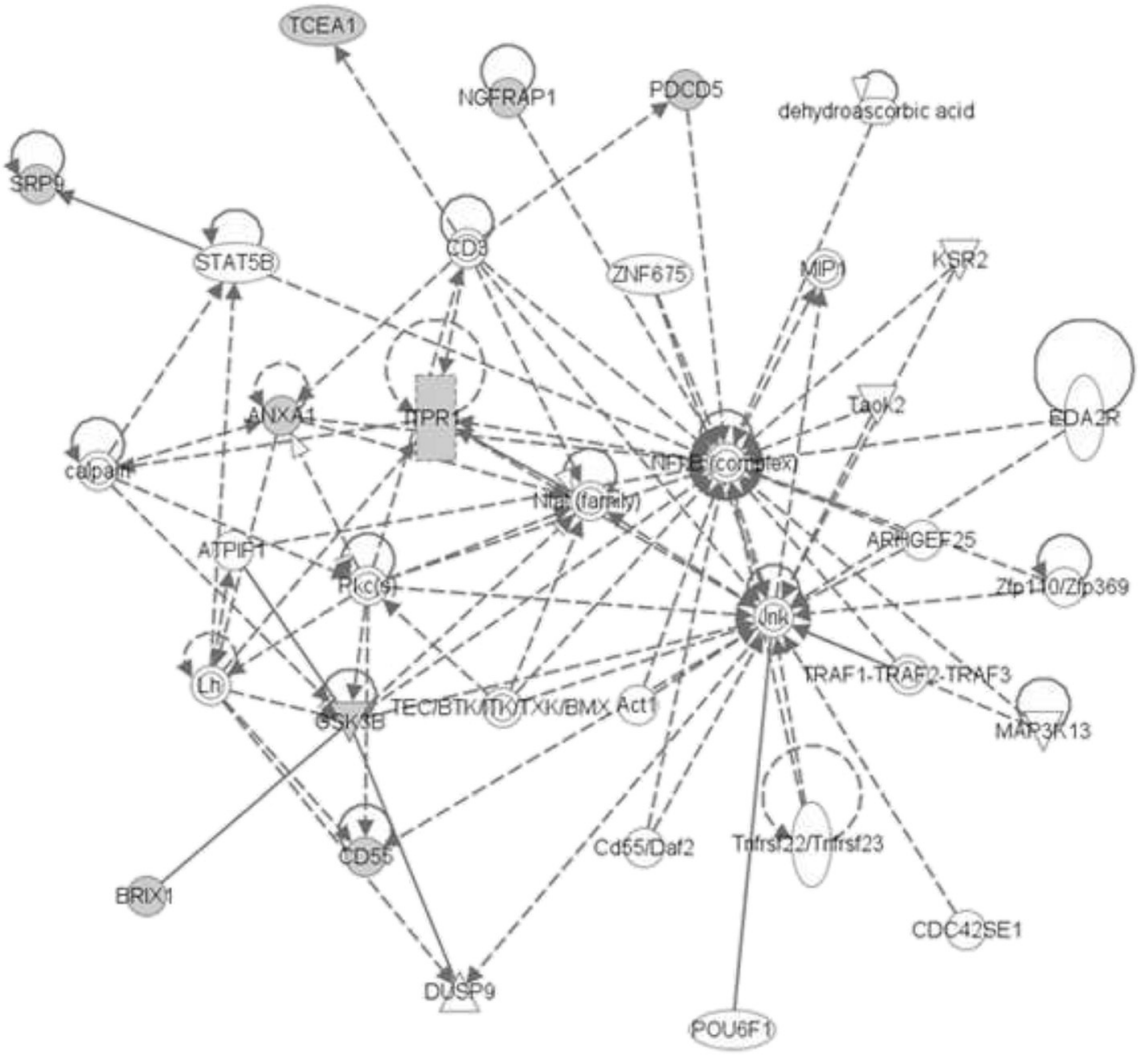
Network of gene interaction *NF-KB* pathway obtained from the subtraction of the control Hep-2 cells. Ingenuity Systems© software reveals a network of gene interactions associated with cancer, squamous cell tumor, benign tumor, apoptosis, proliferation, aggregation, morphology, viability, development and cell signaling, cell cycle control, cell membrane fusion, cleavage RNA fragment, protein transport, angiogenesis, activation of the complement system, innate immune response, release and activation of the calcium ion, inflammatory, antimicrobial response, inherited disorders, organ hypoplasia, oral cancer and laryngeal cancer process

Five differentially expressed genes, *ITPR1, ANXA1, TCEA1, CD55* and *NGFRAP1*, were selected for validation by quantitative real-time PCR experiments. These genes are associated with calcium ion transport, inflammatory response, DNA repair, complement activation and apoptotic process respectively. They were chosen because they are directly or indirectly involved in tumorigenic and inflammatory processes (Table 2 and Fig. 2).

### Real-time PCR validation of differentially expressed genes

**Fig. 3:**
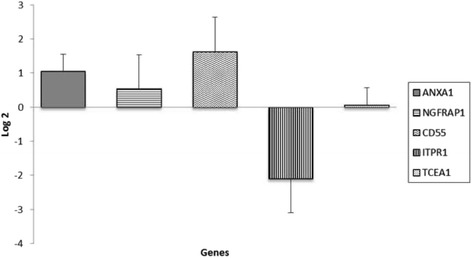
Quantitative Real time PCR gene expression in Hep-2 cells treated with *E. tirucalli latex*. Expression of *ITPR1, ANXA1, TCEA1, CD55* and *NGFRAP1* genes in Hep-2 cells treated with 25 μg/ml of *E. tirucalli* latex compared with control cells. The data represent the logarithm base 2

The RaSH analysis indicated the down-regulation of one gene (*ITPR1*) and up-regulation of four genes (*ANXA1, TCEA1, CD55* and *NGFRAP1*) in Hep-2 cells treated with *E. tirucalli* latex. The quantitative PCR experiment showed that only the expression of *ITPR1* was significantly reduced, however the expression of *CD55* and *ANXA1* genes was significantly increased in Hep-2 cells after treatment with *E. tirucalli* latex (Fig. 3). The expression of other genes oscillated without statistical significance, which was most likely because the *E. tirucalli* does not quantitatively modify the expression of the selected genes despite its interaction with the cell (Additional file 4).

## Discussion

The active fraction present in the *E. tirucalli* latex that was used in our experiments inhibits proliferation of tumor-related enzymes and presents potential anti-inflammatory and analgesic effects [26]. Also, due to the importance of its antitumogenic action, we analyzed the effect of herbal medicine in the cell line of laryngeal squamous cell carcinoma (Hep-2) and it was observed that the morphology did not change after treatment with *E. tirucalli* latex, however, cell proliferation decreased significantly. Other authors corroborate these results, they also observed that treatment with *E. tirucalli* in cell line of breast cancer did not alter the morphology of these cells independent on the vehicle [15]. Regarding the reduced proliferation, *E. tirucalli* must have acted in tumorigenic cells causing cell death and also causing the growth of these cells to be reduced. In vitro*,* it is known that latex works in the cell colonies fighting breast cancer and gastric cancer [19, 27].

The *E. tirucalli* latex effect was also analyzed in the gene expression, for the first time, by RaSH that isolates differentially expressed genes including new genes and rare transcripts as can be seen in some researches [28] and others who use this technology [29]. It is possible to generate significant genomic libraries that can assist in the search for genes differentially expressed in treated Hep-2 cells with herbal cell lines which is faster and less costly than other alternative hybridization techniques [24].

The results showed changes in the expression of some genes in the cells of LSCC after treatment with *E. tirucalli* latex, including *ANXA1*, *TCEA1*, *NGFRAP1*, *ITPR1* and *CD55* genes. These genes belong to the same gene signaling network related by software IPA, correlating the tumorigenic and inflammatory processes. Other authors used the software IPA to identify the biological activities of the genes that distinguish between the thermal properties, hot (warm effects, disperse cold) or cold (cooling effect, removes heat) of Chinese medicinal herb. It was demonstrated that the main biological gene networks in the herbs with hot properties include inflammation and immunity regulation while the genes of the herbs with cold properties affect cell growth, proliferation and development [30].

The selection of the validated *ITPR1* gene (inositol 1,4,5-trisphosphate receptor, type 1) can contribute significantly to the understanding of processes including cancer of the larynx since it has functions related to apoptosis, tumorigenesis of cells squamous and cell proliferation. Inositol 1,4,5-trisphosphate receptor type1 (IP3R1) is known as an IP3-gated Ca^2+^ channel that is located on the endoplasmic reticulum (ER). The ER stores intracellular Ca^2+^ and it has been reported to play a critical role in a variety of cell functions including cell proliferation, contraction of smooth muscle and apoptosis by pumping out Ca^2+^ from ER to cytosol [31–33].

We observed reduced *ITPR1* gene expression in Hep -2 cells after treatment with *E. tirucalli* latex. Other project showed that IP3 receptors act as in vivo substrates for Akt kinase, which plays a key role in suppressing apoptosis [34]. According to our tests, it can be inferred that the *E. tirucalli* latex decreases the expression of *ITPR1* gene by restricting the action of the enzyme Akt kinase and activating apoptosis, decreasing cell growth of LSCC since these receptors are substrates for the action of this enzyme.

Another gene studied was *ANXA1* (Annexin 1), there was increased expression in Hep-2 cells after treatment with *E. tirucalli* latex. This gene is important for the carcinogenic process such as cell cycle control, regulation of apoptosis, cell proliferation and growth, inflammatory response and calcium ion transport regulation [35]. The annexin family consists of ubiquitous proteins that interact with phospholipids in a Ca^2+^-dependent manner. Each annexin consists of an N-terminal domain and a typical core domain. The annexin core is conserved among all members of the annexin family. In contrast, N-terminal domains are variable in length and are believed to regulate annexin functions, forming a truncated molecule with altered sensitivity to calcium [36].

Changes in the expression of *ANXA1* have been reported in various cancers and, furthermore, its expression has been compared to that of other proteins. This scenario prompted Jorge et al. [37] to investigate the relationship between the gene and protein expression of annexin-A1 (*ANXA1*/AnxA1) and galectin-1(*LGALS1*/Gal-1) in an inflammatory gastric lesion as chronic gastritis (CG) and gastric adenocarcinoma (GA) and its association with *H. pylori* infection. High *ANXA1*mRNA expression levels were observed in 90 % of CG cases and in 80 % of GA cases. However, *LGALS1* mRNA levels were high in 60 % of the GA cases, while low expression was found in CG. These results have provided evidence that galectin-1 and mainly annexin-A1 are overexpressed in both gastritis and gastric cancer, suggesting a strong association of these proteins with chronic gastric inflammation and carcinogenesis [37].

The expression of *ANXA1* was evaluated in larynx cancer and it was observed that this protein plays a regulatory role in Hep-2 cells, decreasing the growth of these cells [38]. We infer that the *E. tirucalli* latex contributes to increased expression of the gene *ANXA1* in the larynx squamous cell carcinoma, confirming the role of Annexin A1 in the regulation of cell proliferation, besides the anti-inflammatory function.

The gene *CD55* (decay accelerating factor) presents functions related with squamous tumor cells, apoptosis, cell growth and proliferation, inflammatory response, calcium ion transport regulation and innate immune response, according to the reported data Gene Ontology (GO).

This gene is a key regulator affecting all three complement activation pathways and encoding a globular glycoprotein known as DAF - decay accelerating factor. DAF inhibits the activation of C3 and C5 by preventing the formation of new and accelerating the decay of preformed C3 and C5 convertases which can mediate the downstream effect of all 3 complement-activation pathways, therefore, *CD55* functions to protect host cells from complement attack and inhibits amplification of the complement cascade [39].

*CD55* is an anti-inflammatory and immunological anti-adhesive molecule implicated in the resolution of ongoing inflammation of mucosal epithelia through clearance of transmigrating neutrophils [40]. In our tests, it was demonstrated that the expression of *CD55* was elevated in cell lines of laryngeal squamous cell carcinoma after treatment with *E. tirucalli* latex, confirming its anti-inflammatory role.

The genes evaluated in this study showed differential expression in Hep-2 cells in a manner dependent on *E. tirucalli* latex treatment. This study highlights the importance of the *RaSH* technique as a fast and efficient method for identifying genes modulated by *E. tirucalli* latex in cells of epithelial origin. The functions of the genes identified in this study are important in tumor progression and are amenable to changes through interactions with the metabolism of the Hep-2 cells. The treatment for larynx carcinoma commonly involves surgery, chemotherapy or radiotherapy. However, new therapeutics such as the use of gene therapy associated with nanoparticles tested in larynx cancer derived Hep-2 cells are being developed, allowing the delivery of drugs into target cells.

Furthermore, the *E. tirucalli* latex alters the proliferation and gene expression of Hep-2 cells. These changes are most likely related to inflammatory and tumorigenic processes. Thus, we suggest that the this herb has modulatory effects on the metabolism of this cell type. Our findings suggest the importance of future studies using Hep-2 cells for the development of new therapies. These studies should focus on gene activities that involve the action of *E. tirucalli* and applications of this herb as a possible therapeutic in the treatment larynx carcinoma.

## Conclusions

These findings show that the *E. tirucalli* latex alters the expression of genes with roles in signaling cascades involved in the inflammatory and tumorigenic processes. *E. tirucalli* latex was also shown to alter the proliferation of Hep-2 cells. Thus, our results point to possible applications of this herb as an indicator of innovative therapies for the treatment of LSCC.

### Ethics approval and consent to participate

Not applicable in this section.

### Consent for publication

Not applicable in this section.

## Additional files

Additional file 1: Patente Euphorbia tirucalli. (PDF 96 kb) Additional file 2: Excel spreadsheet about Figure 1C – raw data on cell count. (XLS 41 kb) Additional file 3: Excel spreadsheet about Figure 1D – raw data on cell count. (XLS 23 kb) Additional file 4: Excel spreadsheet about Figure 3 – raw data on quantitative PCR. (XLS 74 kb)

## Abbreviations

ACTB: beta-actin
AKt: protein kinase
ANXA1: annexin A1
APC: adenomatous polyposis coli
BLAST: basic local alignment search tool
C3: C3 convertase enzyme
C5: C5 convertase enzyme
Ca^2+^: calcium
CD55: decay accelerating factor for complement
cDNA: complementary DNA
CG: chronic gastritis
c-myc: myelocytomatosis oncogene
CO_2_: carbon dioxide
CYP1A1: cytochrome P450, family 1, subfamily A, member 1
CYP1B1: cytochrome P450, family 1, fubfamily B, polypeptide 1
CYP2E1: cytochrome P450, family 2, subfamily E, member 1
DAF: decay accelerating factor
DMEs: polymorphisms in drug-metabolizing enzymes
DNA: deoxyribonucleic acid
DNA Pol I: DNA Polymerase I
DNase: deoxyribonuclease
dNTP: deoxynucleotide - solution mix
DTT: DL-Dithiothreito
EDTA: ethylenediaminetetraacetic acid
EGFR: epidermal growth factor receptor
ER: endoplasmic reticulum
FHIT: fragile histidine triad
GA: gastric adenocarcinoma
GAPDH: Glyceraldehyde 3-phosphate dehydrogenase
GC: guanine and cytosine
GO: gene ontology
Hep-2: cells epidermoid carcinoma of the larynx
HNSCC: head and neck squamous cell carcinoma
HPLC: high performance liquid chromatography
HPV: human papilloma virus
H-ras: harvey rat sarcoma virus oncogene homolog
IP3: inositol 1,4,5-triphosphate
IP3R1: inositol 1,4,5-trisphosphate receptor type1
IPA: ingenuity pathway - ingenuity systems©
ITPR1: inositol 1,4,5-trisphosphate receptor, type 1
KCl: potassium chloride
LGALS1/Gal-1: galectin-1
LSCC: larynx squamous cell carcinoma
MboI: enzyme gene from Moraxella bovis
MEM: minimum essential medium
MgCL_2_: magnesium chloride
min: minutes
mL: milliliter
mM: milimolar
mRNA: messenger RNA
NaCl: sodium chloride
NF-κB: nuclear factor kappa-light-chain-enhancer of activated B cells
ng: nanogram
NGFRAP1: nerve growth factor receptor (TNFRSF16) associated protein 1
nM: nanomolar
p16: cyclin-dependent kinase inhibitor 2A
P450: cytochrome P450
pb: base pairs
PCR: polymerase chain reaction
PTEN: phosphatase and tensin homolog
RaSH: rapid subtraction hybridization
Rb: retinoblastoma
RNA: ribonucleic acid
RNase H: ribonuclease H
RT: reverse transcriptase
RT-qPCR: quantitative PCR
SDS: sodium dodecyl sulfate
Sub I: subtraction I - tester = Hep-2 control cells (no treatment) versus driver = Hep-2 cells treated with aveloz
Sub II: subtraction II - tester = Hep-2 cells treated with aveloz versus driver = Hep-2 control cells (no treatment)
T4 DNA Pol: T4 DNA Polymerase
TCEA1: transcription elongation factor A protein 1
TE: buffer Tris-EDTA
TGF-β: transforming growth factor beta
TP53: tumor protein p53
TPA: 12-O-tetradecanoilphorbol-13-acetato
Tris-HCl: hydroxymethyl aminomethane hydrochloride
Trizol: monophasic solution of phenol and guanidine thiocyanate
U: units
XhoI: enzyme gene from Xanthomonas holcicola
μg: microgram
μL: microliters
μM: micromolar

## References

[1] Islami F, Fedirko V, Tramacere I, Bagnardi V, Jenab M, Scotti L, Rota M, Corrao G, Garavello W, Schuz J, Straif K, Negri E, Boffetta P, La Vecchia C (2011). Alcohol drinking and esophageal squamous cell carcinoma with focus on light-drinkers and never-smokers: a systematic review and meta-analysis. Int J Cancer.

[2] Pai SI, Westra WH (2009). Molecular pathology of head and neck cancer: implications for diagnosis, prognosis, and treatment. Annu Rev Pathol.

[3] Hashibe M, Brennan P, Chuang SC, Boccia S, Castellsague X, Chen C, Curado MP, Dal Maso L, Daudt AW, Fabianova E, Fernandez L, Wünsch-Filho V, Franceschi S, Hayes RB, Herrero R, Kelsey K, Koifman S, La Vecchia C, Lazarus P, Levi F, Lence JJ, Mates D, Matos E, Menezes A, McClean MD, Muscat J (2009). Interaction between tobacco and alcohol use and the risk of head and neck cancer: pooled analysis in the International Head and Neck Cancer Epidemiology Consortium. Cancer Epidemiol Biomarkers Prev.

[4] Negri E, Boffetta P, Berthiller J, Castellsague X, Curado MP, Dal Maso L, Daudt AW, Fabianova E, Fernandez L, Wünsch-Filho V, Franceschi S, Hayes RB, Herrero R, Koifman S, Lazarus P, Lence JJ, Levi F, Mates D, Matos E, Menezes A, Muscat J, Eluf-Neto J, Olshan AF, Rudnai P, Shangina O, Sturgis EM (2009). Family history of cancer: pooled analysis in the International Head and Neck Cancer Epidemiology Consortium. Int J Cancer.

[5] Maurya SS, Katiyar T, Dhawan A, Singh S, Jain SK, Pant MC, Parmar D (2014). Gene-environment interactions in determining differences in genetic susceptibility to cancer in subsites of the head and neck. Environ Mol Mutagen.

[6] Jorde LB, Carey JC, Bamshad MJ, White RL (2000). Genética do Câncer. Genética Médica.

[7] Choi S, Myers JN (2008). Molecular pathogenesis of oral squamous cell carcinoma: implications for therapy. J Dent Res.

[8] Dasgupta S, Dash R, Das SK, Sarkar D, Fisher PB (2012). Emerging strategies for the early detection and prevention of head and neck squamous cell cancer. J Cell Physiol.

[9] Gillison ML, D’Souza G, Westra W, Sugar E, Xiao W, Begum S, Viscidi R (2008). Distinct risk factor profiles for human papillomavirus type 16-positive and human papillomavirus type 16-negative head and neck cancers. J Natl Cancer Inst.

[10] Morshed K, Polz-Gruszka D, Szymański M, Polz-Dacewicz M (2014). Human Papillomavirus (HPV) - structure, epidemiology and pathogenesis. Otolaryngol Pol.

[11] Platek ME, Myrick E, McCloskey SA, Gupta V, Reid ME, Wilding GE, Cohan D, Arshad H, Rigual NR, Hicks WL, Sullivan M, Warren GW, Singh AK (2013). Pretreatment weight status and weight loss among head and neck cancer patients receiving definitive concurrent chemoradiation therapy: implications for nutrition integrated treatment pathways. Support Care Cancer.

[12] Franzi SA, Silva PG (2003). Avaliação da qualidade de vida em pacientes submetidos à quimioterapia ambulatorial no Hospital Heliópolis. Revista Brasileira de Cancerologia.

[13] Betancur-Galvis LA, Morales GE, Forero JE, Roldan J (2002). Cytotoxic and antiviral activities of Colombian medicinal plant extracts of the Euphorbia genus. Mem Inst Oswaldo Cruz.

[14] Melo JG, Santos AG, de Amorim EL, Do Nascimento SC, de Albuquerque UP (2011). Medicinal plants used as antitumor agents in Brazil: an ethnobotanical approach. Evid Based Complement Alternat Med.

[15] Aquino CL, Barbosa GM, Barbosa GM, Varricchio MBA, Veiga VF, Kuster RM, Zancan P, Sola-penna M, Holandino C (2008). High dilutions of *Euphorbia tirucalli L.* (aveloz) modify the viability and glycolytic metabolism of cell lines. Int J High Dil Res.

[16] Lirio LG, Hermano ML, Fontanilla MQ (1998). Note antibacterial activity of medicinal plants from the Philippines. Pharm Biol.

[17] Rezende JRR, Rodrigues SB, Jabor IAS, PamphilE JA, Da Rocha CLMSC (2004). Efeito antimutagênico do látex de *Euphorbia tirucalli* no sistema metionina em Aspergillus nidulans. Acta Scientiarum Biol Sci.

[18] Valadares MC, Carrucha SG, Accorsi W, Queiroz ML (2006). *Euphorbia tirucalli L*. modulates myelopoiesis and enhances the resistance of tumour-bearing mice. Int Immunopharmacol.

[19] Santana SS, Gennari-Cardoso ML, Carvalho FC, Roque-Barreira MC, Santiago Ada S, Alvim FC, Pirovani C. Eutirucallin, a RIP-2 type lectin from the latex of *Euphorbia tirucalli L*. presents proinflammatory properties. PLoS One. 2014;9(2):e88422. 10.1371/journal.pone.0088422 PMC3928152 24558388

[20] Lin MW, Lin AS, Wu DC, Wang SS, Chang FR, Wu YC, Huang YB (2012). Euphol from *Euphorbia tirucalli* selectively inhibits human gastric cancer cell growth through the induction of ERK1/2-mediated apoptosis. Food Chem Toxicol.

[21] Passos GF, Medeiros R, Marcon R, Nascimento AF, Calixto JB, Pianowski LF (2013). The role of PKC/ERK1/2 signaling in the anti-inflammatory effect of tetracyclic triterpene euphol on TPA-induced skin inflammation in mice. Eur J Pharmacol.

[22] Pianowski LF, Chaves CP, Calixto JB. Active fraction of a polar solvent extract from the latex of Euphorbiaceae plants. US Patent 2009/0142421 A1; 2009

[23] Sambrook J, Russel DW (2001). Molecular cloning: a laboratory manual.

[24] Jiang H, Kang DC, Alexandre D, Fisher PB (2000). RaSH, a rapid subtraction hybridization approach for identifying and cloning differentially expressed genes. Proc Natl Acad Sci.

[25] Pfaffl MW (2001). A new mathematical model for relative quantification in real-time RT-PCR. Nucleic Acids Res.

[26] Araujo LA, Mrué F, Neves RA, Alves MM, Silva-Júnior NJ, Silva MSB, Melo-Reis PR (2015). Effects of topical treatment with euphorbia tirucalli latex on the survival and intestinal adhesions in rats with experimental peritonitis. Arq Bras Cir Dig.

[27] Kuo PL, Cho CY, Hsu YL, Lin TC, Lin CC (2006). Putranjivain A from *Euphorbia jolkini* inhibits proliferation of human breast adenocarcinoma MCF-7 cells via blocking cell cycle progression and inducing apoptosis. Toxicol Appl Pharmacol.

[28] Rodrigues-Lisoni FC, Peitl Jr P, Vidotto A, Polachini GM, Maniglia JV, Carmona-Raphe J, et al. Genomics and proteomics approaches to the study of cancer-stroma interactions. BMC Med Genomics. 2010;3:1–15. 10.1186/1755-8794-3-14 PMC2881110 20441585

[29] Calmon MF, Rodrigues RV, Kaneto CM, Moura RP, Silva SD, Mota LD, et al. Epigenetic silencing of CRABP2 and MX1 in head and neck tumors. Neoplasia. 2009;11(12):1329–39. 10.1593/neo.91110 PMC2794514 20019841

[30] Liang F, Li L, Wang M, Niu X, Zhan J, He X, Yu C, Jiang M, Lu A (2013). Molecular network and chemical fragment-based characteristics of medicinal herbs with cold and hot properties from Chinese medicine. J Ethnopharmacol.

[31] Lencesova L, Hudecova S, Csaderova L, Markova J, Soltysova A, Pastorek M, Sedlak J, Wood ME, Whiteman M, Ondrias K, Krizanova O (2013). Sulphide signaling potentiates apoptosis through the up-regulation of IP3 receptor types 1 and 2. Acta Physiol (Oxf).

[32] Berridge MJ (1993). Inositol trisphosphate and calcium signalling. Nature.

[33] Demaurex N, Distelhorst C (2003). Apoptosis - the calcium connection. Science.

[34] Khan MT, Wagner L, Yule DI, Bhanumathy C, Joseph SK (2006). Akt kinase phosphorylation of inositol 1,4,5-trisphosphate receptors. J Biol Chem.

[35] Oliani SM, Paul-Clark MJ, Cristian HC, Flower RJ, Perretti M (2001). Neutrophil interaction with inflamed post-capillary venule endothelium alters annexin 1 expression. Am J Pathol.

[36] Gerke V, Moss SE (2002). Annexins: from structure to function. Physiol Rev.

[37] Jorge YC, Mataruco MM, Araújo LP, Rossi AF, de Oliveira JG, Valsechi MC, Caetano A, Miyazaki K, Fazzio CS, Thomé JA, Rahal P, Oliani SM, Silva AE (2013). Expression of annexin-A1 and Galectin-1 anti-inflammatory proteins and mRNA in chronic gastritis and gastric cancer. Mediat Inflamm.

[38] Gastardelo TS, Cunha BR, Raposo LS, Maniglia JV, Cury PM, Lisoni FC, Tajara EH, Oliani SM (2014). Inflammation and cancer: role of annexin A1 and FPR2/ALX in proliferation and metastasis in human laryngeal squamous cell carcinoma. PLoS One.

[39] Lublin DM, Atkinson JP (1989). Decay-accelerating factor: biochemistry, molecular biology, and function. Ann Rev Immunol.

[40] Lawrence DW, Bruyninckx WJ, Louis NA, Lublin DM, Stahl GL, Parkos CA, Colgan SP (2003). Antiadhesive role of apical decay-accelerating factor (*CD55*) in human neutrophil transmigration across mucosal epithelia. J Exp Med.

