# Supplementary material for: Euphorbia tirucalli modulates gene expression in larynx squamous cell carcinoma
Source: BMC Complement Altern Med. 2016 May 21;16:136. doi: 10.1186/s12906-016-1115-z (PMC4875670; doi:10.1186/s12906-016-1115-z)
Supplement: Additional file 1: — Patente Euphorbia tirucalli. (PDF 96 kb) [file 12906_2016_1115_MOESM1_ESM.pdf]

US 20090142421A1

(19) United States

(12) Patent Application Publication

(10) Pub. No.: US 2009/0142421 A1

(43) Pub. Date: Jun. 4, 2009

Pianowski et al.

(54) **ACTIVE FRACTION OF A POLAR SOLVENT EXTRACT FROM THE LATEX OF EUPHORBIACEAE PLANTS**

(75) Inventors: **Luiz F. Pianowski**, Atibaia (BR); **Claudio P. Chaves**, Fortaleza (BR); **Joao B. Calixto**, Florianopolis (BR)

Correspondence Address:
SUGHRUE MION, PLLC
2100 PENNSYLVANIA AVENUE, N.W., SUITE 800
WASHINGTON, DC 20037 (US)

(73) Assignee: **AMAZONIA FITOMEDICAMENTOS LTDA.**, Fortaleza-CE (BR)

(21) Appl. No.: **11/994,228**

(22) PCT Filed: **Jun. 28, 2005**

(86) PCT No.: **PCT/IB2005/001831**

§ 371 (c)(1),
(2), (4) Date: **Apr. 23, 2008**

Publication Classification

(51) **Int. Cl.**
A61K 36/47 (2006.01)
A61K 31/232 (2006.01)
A61P 35/00 (2006.01)

(52) **U.S. Cl.** **424/725**; 514/549

(57) **ABSTRACT**

The present invention generally refers to an active fraction of an extract of the latex of plants from the family Euphorbiaceae in a polar solvent, as well as of one or more compounds contained therein, as well as the use of said fraction and/or said compounds, particularly in the treatment of cancer. The invention also refers to compositions comprising said active fraction and/or said compounds, as well as their use for the treatment of diseases concerning cell proliferation/angiogenesis, particularly cancer.

FIGURE 1

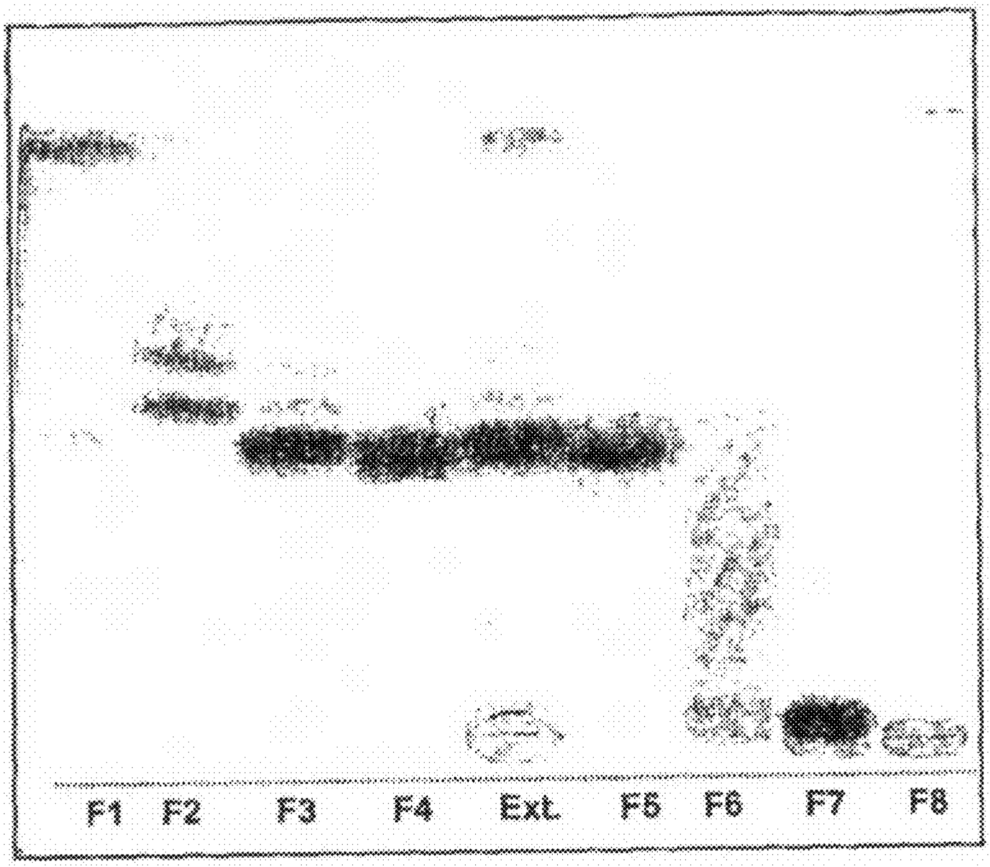

FIGURE 2

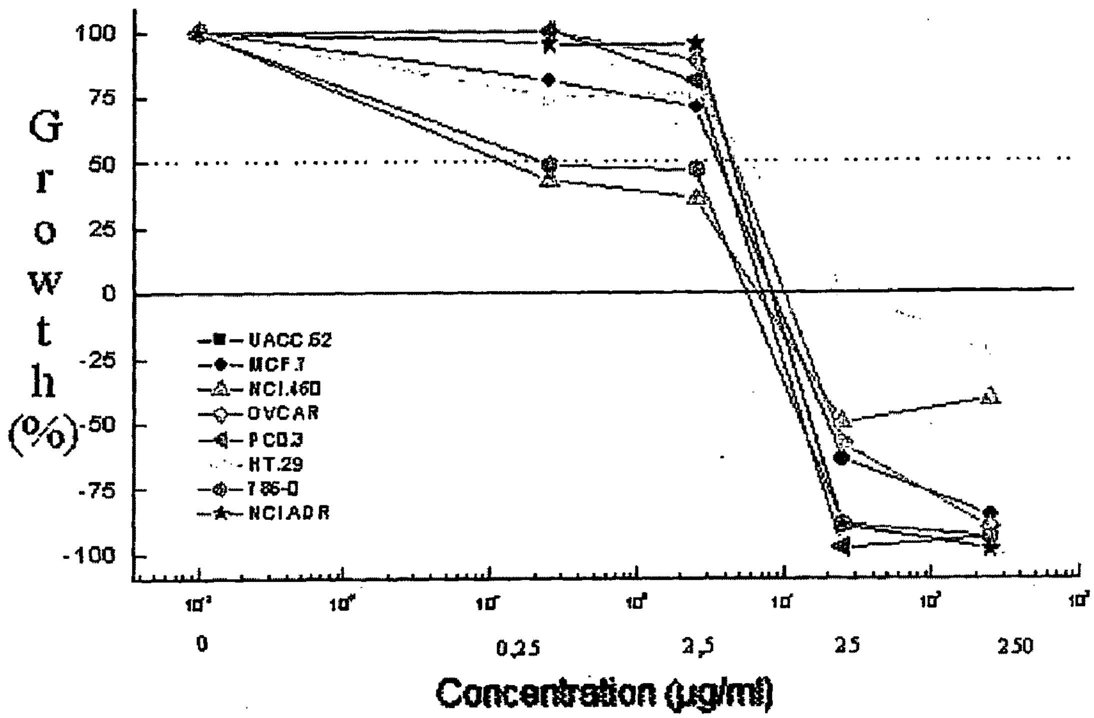
100
75
50
25
0
-25
-50
-75
-100
Growth (%)
UACC 62
MCF.7
NCI.460
OVCAR
PCO.3
HT.29
786-0
NCIADR
0
0,25
2,5
25
250
Concentration (µg/ml)

FIGURE 3

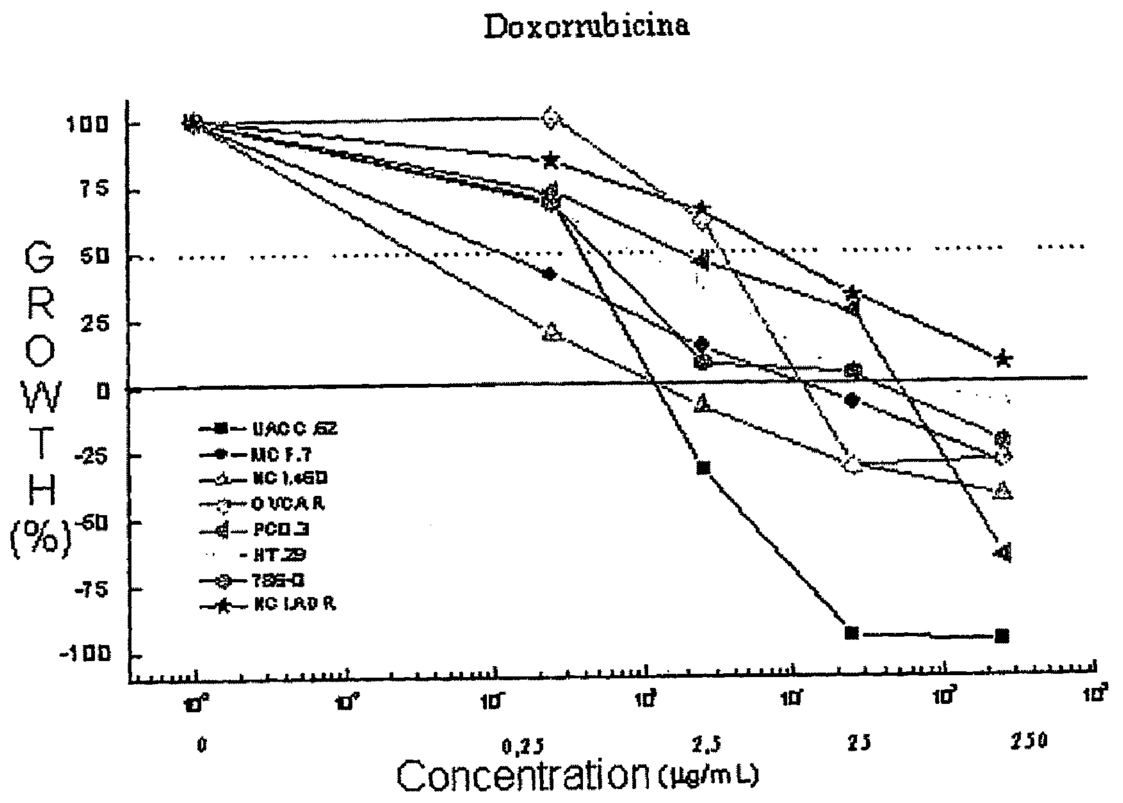
Doxorrubicina
GROWTH (%)
100
75
50
25
0
-25
-50
-75
-100
UACC.62
MCF.7
NCI.460
OVCAR
PCO.3
HT.29
786-0
NCI.ADR
0
0,25
2,5
25
250
Concentration (µg/mL)

ACTIVE FRACTION OF A POLAR SOLVENT EXTRACT FROM THE LATEX OF EUPHORBIACEAE PLANTS

FIELD OF THE INVENTION

[0001] The present invention generally refers to an active fraction of an extract of the latex of plants from the family Euphorbiaceae in a polar solvent, as well as of one or more compounds contained therein, as well as the use of said fraction and/or said compounds, particularly in the treatment of cancer. The invention also refers to compositions comprising said active fraction and/or said compounds, as well as their use for the treatment of diseases concerning cell proliferation/angiogenesis, particularly cancer.

[0002] Particularly, the active fraction of the present invention is obtained from a butanol extract of the *Euphorbia tirucalli Linnaeus* plant latex.

[0003] Particularly, the present invention refers to the use of 3-(2,4,6 dodecatrienoyl)-ingenol and 3-(2,4,6,8-tetradecatetranoyl)-ingenol to obtain a useful composition or medicine for the treatment of diseases concerning cell proliferation, or the treatment of diseases related to cell proliferation.

BACKGROUND OF THE INVENTION

[0004] Cancer, within a particular example of disease involving undesirable cell proliferation or angiogenesis, has deserved more and more studies concerning its combat. Many treatment alternatives have been researched, among them the use of phytomedicine, as in the present invention.

[0005] *Euphorbia tirucalli L.* is a plant from the Euphorbiaceae family, originated from East Africa and Asia, also popularly known as aveloz or pencil tree, milkbush, esqueleto, graveto do cão, figueira do diabo, dedo do diabo, pau-pelado, São Sebastião tree, espinho-de-cristo, coroa-de-cristo, espinho-de-judeu, espinho italiano, pau-sobre-pau, árvore de coral. It is a plant whose parts, e. g. leaves and husk, are used in popular medicine.

[0006] However, many Euphorbiaceae plants, particularly *Euphorbia tirucalli L.,* exude a latex which is toxic, irritating and caustic. Its milky juice may cause damage and edema to skin and mucosa, irritation, eye tearing, eyelid edema and even difficulties in vision. Latex ingestion may also cause nausea, vomiting, diarrhea and, in larger quantities it may even be deadly. In fact, aveloz latex is rich in terpenes, including forbol and ingenol esters. Forbol esters are highly irritating, reported to promote the appearance of tumors (Khan, A. Q. et al, *Euphorcinol: a New Pentacyclic Triterpene from Euphorbia tirucalli, Planta Medica,* 1989; 55: 290-291). A particular aveloz forbol, 4-deoxyforbol ester, was clinically reported as increased the infection of the Epstein-Barr virus (EBV), causing disruptions to the DNA of immune cells and causing suppression of the immune system in general (MacNeil, A. et al, *Activation of Epstein-Barr Virus Lytic Cycle by the Latex of the Plant Euphorbia tirucalli, Br. J. Cancer,* 2003; 88 (10): 1566-9). Besides this chemical compound, an aveloz extract was also reported as having reduced the ability of certain immune cells (T cells) to eliminate EBV. EBV is a member of the herpes virus family, which is one of the most common human viruses. After the initial infection, EBV establishes whole life latent infection within cells B. An EBV infection may cause mononucleosis, and some EBV vehicles will develop cancer, such as Burkitt's lymphoma or nosefaringeous carcinoma. In summary, said latex is aggressive to the human body and therefore seen and recommended as something with which any contact should be avoided.

[0007] Thus, against all technical prejudice, the Applicant verified that a specific active fraction of an extract of said latex, as well as one or more compounds composing it, has effective anticancer action, as will be explained below.

DISCLOSURE OF THE INVENTION

[0008] In an aspect, the object of the present invention is an active fraction of an extract of the latex of Euphorbiaceae plants in a polar solvent. Its preparation process is one of the aspects of the present invention.

[0009] Appropriate polar solvents are the ones known as being of high or medium polarity, particularly those provided with dipole moment between about 1.60 and about 1.80 and dielectric constant between about 15 and about 18. Alcohols such as butanol are particularly appropriate.

[0010] Euphorbiaceae plants which are particularly useful to the present invention are the ones of the *Euphorbia* genus; more particularly, the latex used to obtain an active fraction is from the plant *Euphorbia tirucalli L.*

[0011] Said active fraction presents anticancer activity, as shown by the tests below, which do not limit the scope of the invention, which is determined by the attached claims.

Obtaining an Active Fraction of Butanol Extract of *Euphorbia tirucalli L.*

[0012] A mixture of latex of *Euphorbia tirucalli L.,* preferably fresh, with hexane is made, e. g. 1:1 by weight. Precipitation occurs. The decanted solid fraction (or even its mixture with the liquid fraction) is mixed with n-butanol, preferably under enough agitation to allow effective extraction of the components, as the more polar substances have more affinity with butanol, while less polar substances have more affinity with hexane.

[0013] Separation of the butanol fraction (by HPLC, liquid-liquid chromatography, column chromatography or equivalent means) allows the compounds present therein to be taken off in group scales, mainly by size. In a column chromatography separation with silica gel Sephadex G75, using a mixture of hexane: ethyl acetate (0% to 100%), eight fractions are separated from the butanol fraction of the latex extract.

[0014] The obtained fractions, in this separation privileging fractioning by molecular weight, were submitted to thin layer chromatography with silica gel, with a 8:2 mixture by weight of hexane: ethyl acetate. The chromatogram as obtained is shown by FIG. **1**.

[0015] It can be seen that fraction 7 is well defined, indicating the sure presence of components with higher polarity, with solubility and affinity characteristics with the substrate. As a person skilled in the art knows, fractions 6 and 8 may also contain minor quantities of the components found in fraction 7, due to characteristics of the fractioning method itself.

[0016] Said fraction 7 was submitted to tests to verify its anticancer action, as disclosed below.

EXAMPLES

In Vitro Model to Evaluate the Antiproliferation Activity of Human Tumor Cell Lines by Using the Sulforhodamine B Assay.

[0017] To perform the test, cancer cell lines MCF-7, NCl-ADR, OVCAR -03, PC 03, 786-0 and HT-29 were selected,

cultivated in RPMI/SFB (RPMI refers to RPMI 1640—Roswell Park Memorial Institute cultivation medium, as per *J. Surg. Oncol.* 1969; 1 (2); 153-66; SBF refers to inactivated bovine fetal serum) with 5% SFB; the mentioned cell lines were supplied by the National Cancer Institute NCl, United States of America (Table 2).

TABLE 2

Cell panel to evaluate antiproliferation activity.

Type of cell	Code	Type of culture
Lung	NCI460	Adhered
Breast	MCF-7 NCI ADR*	Adhered
Colon	HT 29	Adhered
Kidney	786-0	Adhered
Ovary	OVCAR-3	Adhered
Prostate	PC-3	Adhered

*Cell line expressing resistance phenotype to multiple drugs.

Cells are kept in 25 cm^2 flasks with 5 ml of RPMI/SFB at 37 ° C. under 5% CO_2 and 100% humidity atmosphere, replicated whenever the formed carpet reaches about 80% confluence.

Assay to Determine the Antiproliferation Activity of Assayed Substances.

[0018] 100 μl of cells in RPMI/SFB/gentamicin are inoculated under their corresponding inoculation densities (pre-established through growth curves) in 96-well plates.

[0019] After 24 hours of incubation at 37° C. in 5% CO_2 and 100% humidity atmosphere, the assay substance (0.25 to 250 μg/ml) in 100 μg/ml volume is added. At that moment, a control plate is fixed to determine the absorbency at the moment of addition of the assay substance (value T_0—represented in the attached graph by the full line on point zero). After 48 hours of incubation, the other plates will be fixed to determine the protein content.

Sample Dilution

[0020] To produce stock solutions, samples are diluted in sodium dimethylsulfoxide (DMSO) in 100 mg/ml concentration. For addition to the experimental plates, those solutions are diluted 400 times in RPMI/SFB/gentamicin.

Colorimetric Assay with Sulforhodamine B (SRB)

[0021] This assay is run according to Skehan et al,—*New Colotimetiic Cytotoxicity Assay for Anticancer-Drug Screening. J Natl Cancer Inst* 82: 1107-1112 (1990).

[0022] After 48 hours of incubation, cells are fixed with 50 μl of trichloroacetic acid (TC) at 50% at 4° C. To complete cell fixation, plates will be incubated for one hour at 4° C.

[0023] After being fixed with trichloroacetic acid, plates are submitted to four washes with distilled water to remove TCA residues, cultivation medium, bovine fetal serum and secondary metabolites, and subsequently kept at room temperature until fully dried.

[0024] Plates are then colored by adding 50 μl of sulforhodamine (SRB) at 0.4% (weight/volume) dissolved in 1% acetic acid and incubated for 30 minutes at 4° C. They are then washed for four consecutive times with 1% acetic acid. The residue of the washing solution is removed and the plates are again dried at room temperature. The coloring agent linked to cell proteins is solubilized with tris(hydroxymethyl) aminornethane buffer (Trizma base®, supplied by Sigma Aldrich Fine Chemicals, U.S.A.), with 10 μmM concentration and pH 10.5 for five minutes in ultrasound. Spectrophotometric reading of absorbency is achieved with 560 nm in an ELISA reader.

Result Analysis

[0025] The average absorbencies discounted from their respective blanks are calculated and the growth inhibition (GI) of each assayed samples is determined with the help of the formula below. Results obtained are analyzed, considering that:

[0026] if T>C, cell growth was stimulated;

[0027] if $T \leqq T_0$ but<C, there was cytostatic activity (growth inhibition) and the used formula is $100\times[(T-T_0)/(C-T_0)]$;

[0028] if $T<T_0$, there was cytocidal activity (cell death) and the used formula is $100\times[(T-T_0)/(C-T_0)]$;

[0029] wherein T is the average absorbency of the treated cell, C is the cell control and T_0 is the control of cells on the day of addition.

[0030] Finally, it is also possible to subtract the obtained result from 100%, thus obtaining the growth inhibition (GI) percentage.

[0031] The graphs on FIGS. **2** and **3** respectively present the concentration/activity curves for the assayed active fraction and for doxorhubicine, a chemotherapeutical product used as a positive control for cells in different concentrations (250 to 0.25 μg/ml), relating the percentage of cell growth and the concentration of the utilized extract.

[0032] Samples are considered as active when they present growth inhibition of more than 50% (represented in the graph by the line on point 50) in a concentration-dependent fonm and preferably presenting cell selectivity (different activity between the cell lines or specific activity for one of the cell lines).

[0033] IC50 values (concentration inhibiting 50% growth) were determined by sigmoidal non-linear regression, using the analysis of the GraphPad Prism software (from the company GraphPad Software Inc., San Diego Calif., U.S.A.); doxorhubicine is the positive control.

[0034] FIG. **2** proves, per se and in comparison with the positive control, the efficacy of the active fraction of an extract of the latex from the Euphorbiaceae plant in a polar solvent, in this case n-butanol, in anticancer activity.

[0035] Within another aspect of the invention, a few particular compounds of molecular weight between about 500 and about 600, specifically detected as components of said active fraction, are themselves provided with anticancer activities.

[0036] The invention also refers to said compounds and their use, solely or in combination among themselves or with others, for the treatment of diseases associated to proliferative cells, particularly cancer, and their use to obtain compositions and medicines used to treat said diseases.

[0037] The following are particularly useful among said compounds with molecular weight between about 500 and about 600, with their corresponding spatial structures:

Compound 1: 3-(2,4,6-dodecatrienoyl)-ingenol (molecular weight 524)

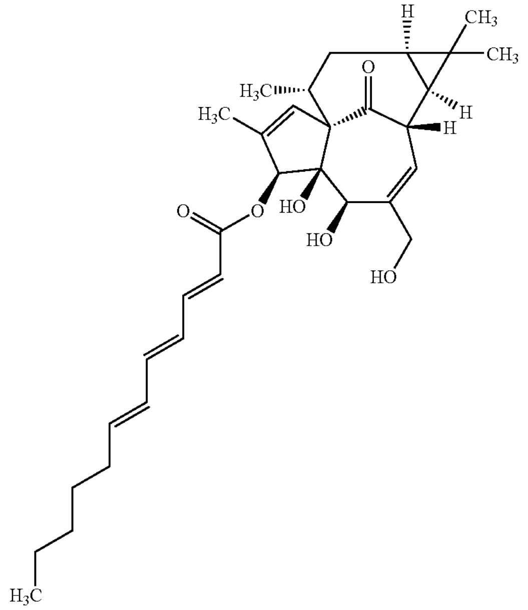

Compound 2: 3-(2,4,6,8-tetradecatetranoyl)-ingenol (molecular weight 550)

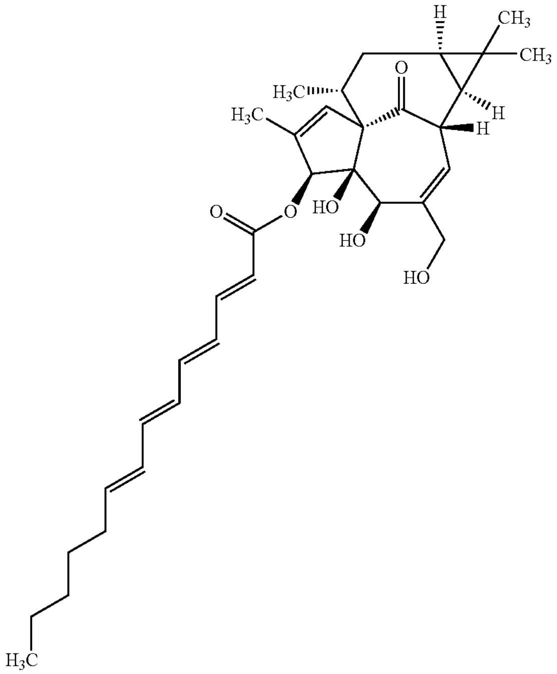

[0038] In an additional aspect of the invention, it encompasses pharmaceutical compositions comprising a pharmaceutically active amount of a fraction of an extract in a polar solvent, particularly n-butane, of the latex of an Euphorbiaceae plant, particularly *Euphorbia tirucalli L.,* jointly with pharmaceutically acceptable excipients.

[0039] The compositions of the invention may contain about 0.001% to about 95% of the Euphorbiaceae latex extract active fraction obtained as previously disclosed.

[0040] In another aspect of the invention, pharmaceutical compositions comprising effective quantities of one or more compounds with molecular weight between about 500 and about 600 as contained in the Euphorbiaceae latex extract active fraction obtained as disclosed, and pharmaceutically acceptable excipients are contemplated.

[0041] In another aspect of the invention, it also encompasses pharmaceutical compositions comprising effective quantities of one or more compounds 1 and 2 as mentioned above and pharmaceutically acceptable excipients.

[0042] Pharmaceutically acceptable excipients to make the compositions of the present invention may include all those known in the art, such as lactose, starch, sucrose, glucose, methyl cellulose, magnesium stearate, dicalcium phosphate, calcium sulphate, mannitol, sorbitol, ethanol, glycerol, water and other ones. A reference work for the formulation of said pharmaceutical forms is the book *Remington's Pharmaceutical Sciences,* from the U. S. publisher Mack Publishing.

[0043] An adequate amount, not excluding any other, of one or more compounds with molecular weight between about 500 and about 600 as mentioned, particularly one or more of the compounds 1 and 2 in the compositions of the invention, is between about 0.1 mg to 2000 mg, particularly between about 10 mg and about 100 mg.

[0044] The compositions of the present invention may be administered orally (including immediate and/or controlled release forms), parenterally (intramuscular; endovenous, intra-arterial, intraperitoneal, intrathecal, subcutaneous or hypodermal and intradermal), via mucosa (lung, sublingual, nasal, conjuntival, rectal, vaginal) and topically.

[0045] Appropriate presentation form for the compositions of the invention, with no limitation, are: solution, syrup, elixir, suspension, emulsion, lotion, ointment, cream, paste, gel, aerosol, powder, pellet, tablet, caplet, suppository, ovule or eye drops.

[0046] The compositions of the invention may also contain, besides the active fraction of the Euphorbiaceae latex polar solvent extract and/or one or more compounds with molecular weight between about 500 and about 600 contained therein, and/or one or more of compounds 1 and 2 as mentioned above, other active principles useful against the type of proliferative cell whose combat is desired. The person skilled in the art knows how to decide on the addition of other known active principles.

[0047] Within another embodiment of the invention, there is a method to treat diseases related to proliferative cells, particularly cancer, in which a patient in need of said treatment receives an effective amount of:

[0048] (1) an active fraction of the latex of the Euphorbiaceae plant extracted with a polar solvent, and/or

[0049] (2) one or more compounds with molecular weight between about 500 and about 600 contained in said active fraction, and/or

[0050] (3) one or more of compounds 1 and 2 as mentioned above, or

[0051] (4) a composition containing any of the preceding or their combinations.

[0052] Within one more aspect of the invention, there is the use of an active fraction of the latex of the Euphorbiaceae plant, particularly *Euphorbia tirucalli L.* extracted with a polar solvent, particularly butanol, characterized by the fact it

is in the preparation of a useful composition or medicine for the treatment of diseases related to proliferative cells, particularly cancer. The present invention also includes the use of one or more compounds with molecular weight between about 500 and about 600 as contained in said active fraction, characterized by the fact that it is in the preparation of a useful composition or medicine for the treatment of diseases related to proliferative cells, particularly cancer. The invention also includes the use of one or more compounds 1 and 2 as mentioned above characterized by the fact that is to prepare a useful composition or medicine for the treatment of diseases related to proliferative cells, particularly cancer.

[0053] The invention is related to proliferative cells of any animal, particularly human beings.

[0054] The person skilled in the art is able to find out other equivalent means to work the present invention from the teachings and examples presented in this document, without departing from the limits set out in the claims as disclosed further below.

1. Active fraction of a polar solvent extract of an Euphorbiaceae plant latex characterized by the fact that it comprises, among eight fractions obtained in separation by chromatography column, the heavier fractions 6, 7 and 8.

2. The fraction of claim **1**, characterized by the fact that it only comprises fraction 7.

3. The fraction of claim **1**, characterized by the fact that said latex is preferably fresh.

4. The fraction of claim **1**, characterized by the fact that said polar solvent has a dipole moment between about 1.60 and about 1.80 and dielectric constant between about 15 and about 18.

5. The fraction of claim **4**, characterized by the fact that said polar solvent is an alcohol.

6. The fraction of claim **4**, characterized by the fact that said solvent is n-butanol.

7. The fraction of claim **1**, characterized by the fact that the Euphorbiaceae plant is from the genus *Euphorbia*.

8. The fraction of claim **7**, characterized by the fact that the plant is *Euphorbia tirucalli L*.

9. The fraction of any of claims **1** to **8**, characterized by the fact that it contains molecules with molecular weight between about 500 and about 600.

10. The fraction of any of claims **1** to **8**, characterized by the fact that it contains one or more of the compounds 3-(2,4,6-dodecatrienoyl)-ingenol and 3-(2,4,6,8-tetradecatetranoyl)-ingenol.

11. Use of an active fraction of a polar solvent extract of an Euphorbiaceae plant latex of any of claims **1** to **10**, characterized by the fact that it is to obtain a useful composition or medicine for the treatment of diseases related to cell proliferation.

12. Use of one or more of 3-(2,4,6-dodecatrienoyl)-ingenol and 3-(2,4,6,8-tetradecatetranoyl)-ingenol, characterized by the fact ft to obtain a useful composition or medicine for the treatment of diseases concerning cell proliferation.

13. A composition containing an effective amount of said active fraction of any of claims **1** to **10**, or one or more of 3-(2,4,6-dodecatrienoyl)-ingenol and 3-(2,4,6,8-tetradecatetranoyl)-ingenol, and pharmaceutically acceptable excipients.

14. The composition of claim **13**, characterized by the fact that it contains about 0.001% to about 95% of said active fraction of one of claims **1** to **11**.

15. The composition of claim **13**, characterized by the fact that it contains about 0.1 mg to 2000 mg, more particularly about 10 mg to about 100 mg, of one or more of 3-(2,4,6-dodecatrienoyl)-ingenol and 3-(2,4,6,8-tetradecatetranoyl)-ingenol.

16. Use of the composition of claim **13**, characterized by the fact that it is to obtain a useful composition or medicine for the treatment of diseases related to cell proliferation.

17. Use of any of claims **11** or **12**, characterized by the fact that said disease is cancer.

18. The use of claim **17**, characterized by the fact that the cancer is lung, breast, colon, kidney, ovary or prostate cancer.

19. A method of treatment of diseases related to proliferative cells, characterized by the fact that a patient in need of said treatment receives an effective amount of an active fraction of any of claims **1** to **10** and/or one or more compounds with molecular weight between about 500 and about 600 contained in an active fraction of any of claims **1** to **10** and/or one or more of 3-(2,4,6-dodecatrienoyl)-ingenol and 3-(2,4,6,8-tetradecatetranoyl)-ingenol, or a composition comprising an effective amount of an active fraction of any of claims **1** to **10** and/or one or more compounds with molecular weight between about 500 and about 600 contained in an active fraction of any of claims **1** to **10**, and/or one or more of 3(2,4,6-dodecatrienoyl)-ingenol and 3-(2,4,6,8-tetradecatetranoyl)-ingenol.

20. The method of claim **19**, characterized by the fact that said disease is cancer.

21. The method of claim **20**, characterized by the fact that the cancer is lung, breast, colon, kidney, ovary or prostate cancer.

22. A process to obtain an active fraction of a polar solvent extract of an Euphorbiaceae plant latex, characterized by the fact that it comprises:

a) mixing latex from an Euphorbiaceae plant with a substantially non-polar solvent;
b) addition of a substantially polar solvent to the mixture of item a) or to the decanted solid phase after mixing item a), under enough agitation to allow effective contact between the solvent and itself;
c) separation of the polar phase;
d) fractioning of the polar phase in a chromatographic column;
e) isolation of the fraction corresponding to higher molecular weights.

23. The process of claim **22**, characterized by the fact that one or more of the following limitations are used:

latex is fresh;
the plant is *Euphorbia,* particularly *Euphorbia tirucalli L.;*
the substantially non-polar solvent is hexane;
the polar solvent has a dipole moment between about 1.60 and about 1.80 and dielectric constant between about 15 and about 18;
the stationary phase of the chromatographic column is Sephadex G75 and dragging is made with a mixture of hexane: ethyl acetate;
the polar phase is fractioned in eight fractions, so that the isolation of the active fraction with higher molecular weights is made with fractions 6, 7 and 8, preferably just fraction 7, more preferably molecules with molecular weight between 500 and 600.

* * * * *
